# Postmastectomy-Postirradiation Atypical Vascular Lesion of the Skin: Report of 2 Cases

**DOI:** 10.1155/2012/710318

**Published:** 2012-09-19

**Authors:** P. S. Jayalakshmy, Aswathy P. Sivaram, Joy Augustine, P. Bindu

**Affiliations:** Department of Pathology, Government Medical College, Kerala, Thrissur 680596, India

## Abstract

The spectrum of vascular lesions developing in breast or chest wall skin following lumpectomy or mastectomy and radiation is wide and ranges from atypical vascular lesions with a benign clinical behaviour to frankly malignant, angiosarcoma ranging histologically from well to poorly differentiated variety. Postmastectomy-postirradiation atypical vascular lesions (AVLs) are rare and develop in the skin adjacent to the mastectomy scar. About hundred cases have been reported in the literature so far. AVLs have also been reported in patients after breast conservation surgery within the breast parenchyma or in the skin around the scar. The incidence appears to be rising. The exact reason for this is not known. The newer modalities of radiation therapy may be contributory to the pathogenesis. More studies have to be done in this area to prove the causal relationship. We are reporting the cases of 2 patients with carcinoma of breast who developed postmastectomy-postirradiation atypical vascular lesions. The cases were received in our department within a 6-month period.

## 1. Introduction

It is frequently difficult to classify the postradiation vascular lesions accurately and they create an emerging diagnostic and therapeutic challenge to both pathologists and clinicians [1]. Atypical Vascular lesion (AVL) of skin after radiation therapy for carcinoma of breast is uncommon. About a hundred cases have been reported in the literature so far. Clinical and histological pictures in these cases do not warrant a diagnosis of angiosarcoma because of lack of features of malignancy. Hence, AVLs are categorized as benign lesions. However there is significant clinical as well as histological overlap. Brenn and Fletcher studied 42 cases of AVLs and concluded that AVLs are part of a continuum and are in fact precursors to angiosarcomas [2].

The lesion may develop within a few months to years after radiation therapy and present as asymptomatic single or multiple macules, papules, or nodules on the skin. They are seen in and around the scar of lump excision or mastectomy, usually in the field of radiation. AVLs have been reported in cases of breast conservation surgery also. We report 2 cases of AVLs, both developed in the chest wall skin of patients who had undergone modified radical mastectomy and received irradiation for carcinoma of breast. The lesions were situated in and around the scar and extended to the adjacent skin and back.

## 2. Case Presentation

### 2.1. Case 1

**Figure 1:**
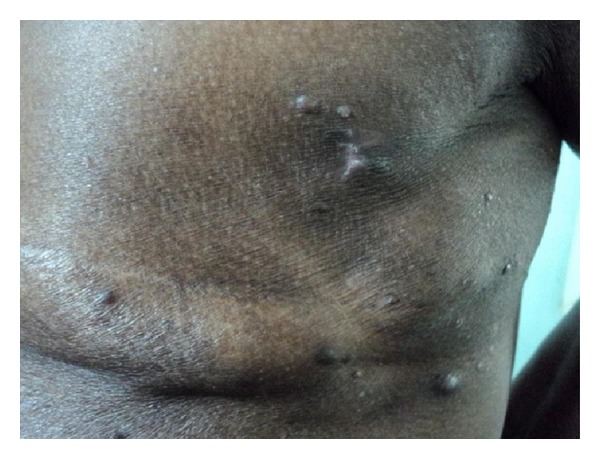
Multiple skin papules and nodules around the mastectomy scar—Case 1.

50-year-old female, diagnosed to have infiltrating duct carcinoma of breast in 2008, underwent modified radical mastectomy (MRM) and radiation therapy. Local irradiation therapy was given. In 2012, the patient presented with multiple papules and nodules on the skin around the MRM scar. The lesions appeared during the past one year at variable interval, some showing spontaneous regression. On examination, multiple nontender and nonitchy papules and nodules were present, with the largest measuring 0.6 cms. The lesions were seen in the chest wall skin near the scar and extending to the adjacent area. A few lesions were seen in the back also (Figure 1).

**Microscopy**

**Figure 2:**
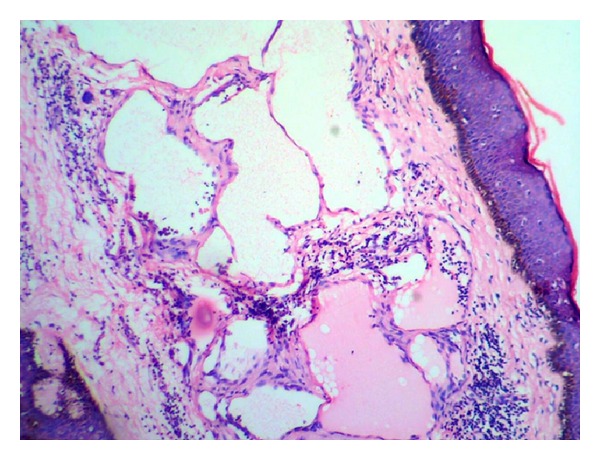
Irregular thin-walled vascular channels of variable sizes in the dermis. Lymphocytes are seen within and outside the lumen (Case 1—H&E ×100).

**Figure 3:**
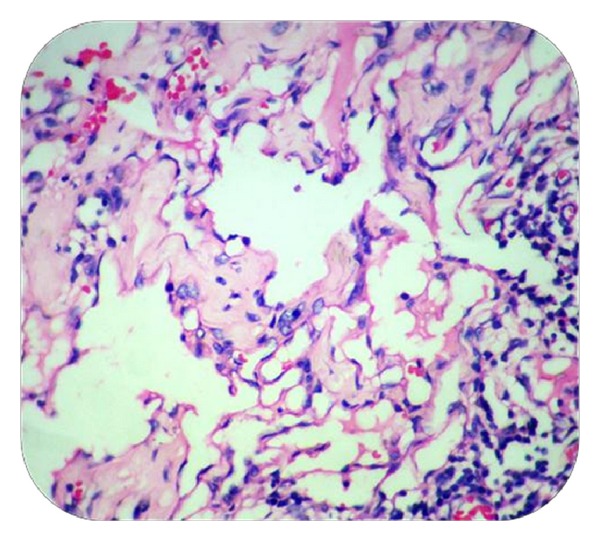
Vascular channels lined by flattened to cuboidal cells. Lymphocytes and RBCs are seen within and outside the lumen (Case 1—H&E ×400).

Sections from the skin nodule showed acanthotic epidermis. Superficial dermis showed focal collections of irregular vascular/lymphatic channels of varying sizes, lined by single layer of endothelial cells without atypia. There were tiny papillary projections into the vascular lumen. RBCs, lymphocyte, and eosinophilic material were seen in some of the lumina. Chronic inflammatory cells were seen infiltrating the dermis. Lesions were confined to papillary and reticular dermis (Figures 2 and 3).

### 2.2. Case 2

**Figure 4:**
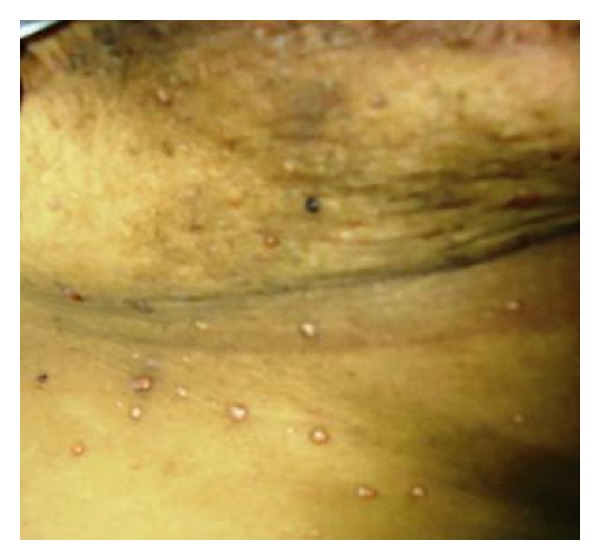
Papules and nodules in and around the mastectomy scar—Case 2.

42-year-old female was diagnosed to have Infiltrating duct carcinoma of breast in 2008. She underwent modified radical mastectomy (MRM) and radiation therapy. 2 years later, in 2010, she noted multiple skin nodules around the scar of MRM. On examination, the lesions were papulo nodular, non tender & non itchy, largest measuring 0.7 cms (Figure 4).

**Microscopy**

**Figure 5:**
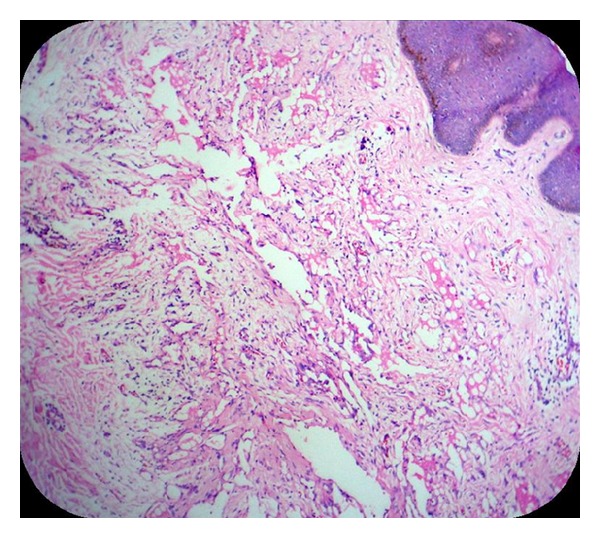
Epidermis with underlying dermis showing thin-walled vascular channels containing RBCs in the lumen (Case 2—H&E ×100).

**Figure 6:**
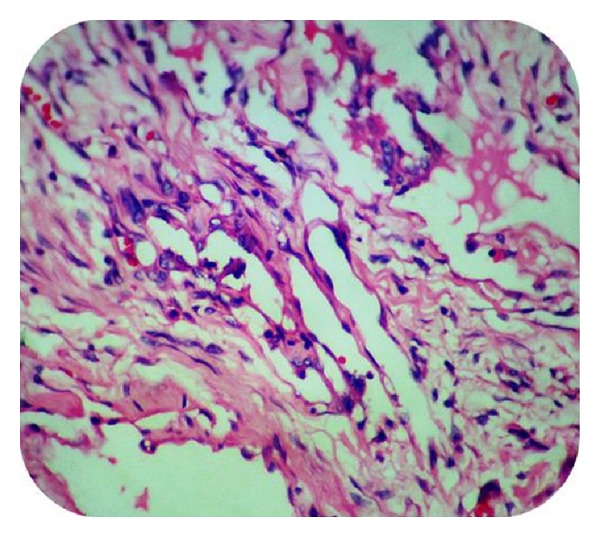
Dermis shows thin-walled vascular channels lined by endothelial cells showing mild atypia. Small papillary projections are seen (Case 2—H&E ×400).

Sections from the skin nodule showed acanthotic epidermis. Underlying dermis showed focal collection of irregular anastomosing vascular channels lined by single layer of endothelial cell with mild cellular atypia. Lesion extended into the reticular dermis. Lumens showed a few lymphocytes, eosinophilic material, and a few RBCs. The rest of the dermis showed perivascular lymphocytic infiltrate and collagenisation (Figures 5 and 6).

## 3. Discussion

Benign vascular lesions arising in the skin of the chest wall following mastectomy and radiotherapy were reported in 1968 by Kurwa and Waddington as “postmastectomy lymphangiomatosis” and in 1978 by Prioleau and Santa Cruz as “Lymphangioma circumscriptum” [3]. In 1994, Fineberg and Rosen described 4 women with cutaneous vascular proliferations after lumpectomy and radiation for breast carcinoma and coined the term “atypical vascular lesion” [4]. Most AVLs pursue a benign course. But there are rare reports of progression to angiosarcoma, usually after multiple recurrences suggesting that AVL may be a precursor to or incipient angiosarcoma [5]. AVLs typically develop 2 to 5 years after radiotherapy, but intervals of a decade or longer have been reported [3]. Lesions present as single or multiple pink papules in the skin measuring 5 mm or less in diameter and are rarely present in breast parenchyma [3]. Although the specific type, technique, and dosage of prior radiation have not been specifically analyzed, all AVLs are observed within the radiation field [6].

Two histologic types of AVL have been described—(1) lymphatic type (LT) in which predominantly thin-walled, variably anastomosing lymphatics are seen primarily in superficial dermis and (2) vascular type (VT) which is composed of predominantly small, irregularly dispersed, capillary-type vessels, invested by pericytes, often blood filled, located in superficial or deep dermis. Vascular type may be associated with extravasated red blood cells or hemosiderin, with minor lymphatic-type component.VT-AVL is found to have highest risk for progression to angiosarcoma [5].

Angiosarcoma accounts for only 1% of soft tissue sarcomas, but it is a rare complication of radiotherapy, with a cumulative incidence of 0.9 per 1000 cases during 15 years [2], with overall 5-year survival of just 16% [7]. Differentiation of AVL from Angiosarcoma is important. Size is especially important in diagnosis since most AVLs are small (median size—0.5 cm), while angiosarcomas are usually much larger (median size—7.5 cm) [2]. As per Fineberg and Rosen's histologic criteria of assessment in their study, AVLs typically lack multilayering of endothelial cells, prominent nucleoli, mitoses, and hemorrhage. In AVL, there will not be destruction of adnexa or extension into the subcutaneous tissue [4]. All the features characteristic of angiosarcoma were absent in both of our cases. Both patients had almost similar clinical presentation and histological appearance. 

 Immunohistochemically, the lining cells of both AVL and angiosarcoma show positivity for CD31 and variable positivity for D2-40, CD34. In our case, the lining cells were positive for CD 31. MYC immunostain is helpful to differentiate between AVL and angiosarcoma. AVL is negative for MYC, whereas postirradiation angiosarcoma shows strong positive nuclear staining for MYC [8].

Regarding treatment, AVLs are not well-known entities and currently lack official prognostic factors and proper guidelines for surgical treatment [6]. For practical purposes, complete excision of postradiation atypical vascular lesions and close follow-up of affected patients are mandatory [8]. But, in our patients, complete excision was not feasible since the lesions were spread over a considerable area in the skin extending to the back. These two patients are now put in close followup and are doing fine with spontaneous regression of some of the papules. Long-term followup of such patients will be necessary to fully characterize the prognostic importance of atypical vascular lesions.

## 4. Conclusion

Though, now considered as a benign lesion, the exact biological behavior of AVLs is not fully established because of the limited number of cases encountered. More cases of post radiation AVLs may be encountered in the future and hence breast carcinoma patients who have received radiation therapy have to be closely monitored for development of papulonodular skin lesions. Long-term follow-up study of these patients is necessary to assess the exact behavior of the lesion and risk of progression to angiosarcoma. The pathogenetic factors have to be elucidated by studying these group of patients.
